# What drives regional variation in hospital reform implementation? Insights from more than a decade of Swiss hospital planning

**DOI:** 10.1016/j.hpopen.2026.100190

**Published:** 2026-10-14

**Authors:** Carla Walker, Charlotte Lang, Daria Bukanova-Berend, Katharina Lonnes, David Ehlig, Justus Vogel, Dr. Alexander Geissler

**Affiliations:** a Chair of Health Economics, Policy and Management, School of Medicine, University of St. Gallen, St.-Jakob-Strasse 21, St. Gallen, 9000, Switzerland; b University of St. Gallen, Dufourstrasse 50, St. Gallen, 9000, Switzerland

**Keywords:** Hospital planning, Hospital capacity planning, Hospital reform, Health policy implementation, Health policy triangle

## Abstract

**Background:**

Implementing national hospital reforms is challenging in decentralised health systems, where subnational governments are responsible for execution. In 2012, Switzerland introduced a national hospital reform, with implementation delegated to cantons. By applying the Health Policy Triangle, we examine how cantons have implemented the national hospital planning reform and what factors explain observed variation.

**Methods:**

We employed a mixed-methods design. A document analysis and 25 semi-structured interviews with experts and cantonal representatives were combined with an unsupervised cluster analysis, examining whether structurally similar cantons showed comparable patterns of deviation.

Findings.

By 2024, 25 out of 26 cantons had adopted service groups with varying introduction timepoints and diffusion toward a shared model. Differences in the number of service groups and most requirements were minor. In contrast, minimum volume requirements varied considerably, driven by political feasibility concerns and the perceived risk of financially destabilising local hospitals.

**Conclusion:**

The Swiss case shows that voluntary harmonisation can emerge in decentralised health systems through policy diffusion, where one canton's planning approach became a de facto standard for others. However, convergence is incomplete where implementation decisions are politically sensitive and where administrative capacities are limited. Decentralised systems may benefit from stronger national standardisation combined with transparent mechanisms for local adaptation, enabling a balance between quality, cost, and access considerations.

## Highlights

• Cross-country learning on regional heterogeneity in reform implementation is limit.
• Mixed methods: document review, 25 interviews, and exploratory clustering.
• Cantons diffuse on methods but differ in politically sensitive requirements.
• Minimum volume requirements show the greatest variation across cantons.
• Findings support balancing national standards with transparent mechanisms for local adoption.

## Glossary

HP: Hospital planning
IVHSM: Interkantonale Vereinbarung über die hochspezialisierte Medizin (English: Inter-cantonal Agreement on Highly Specialised Medicine)
KVG: Bundesgesetz über die Krankenversicherung (English: Federal Law on Health Insurance)
KVV: Verordnung über die Krankenversicherung (English: Ordinance on Health Insurance)
MVR: Minimum volume requirements
SPLB: Spitalplanungs-Leistungsbereiche (English: Hospital Planning Service Areas)
SPLG: Spitalplanungs-Leistungsgruppen (English: Hospital Planning Service Groups)

**Glossary of Swiss cantons**
AG: Aargau
AI: Appenzell Inner-Rhodes
AR: Appenzell Outer-Rhodes
BE: Bern
BL: Basel-Land
BS: Basel-Stadt
FR: Fribourg
GE: Geneva
GL: Glarus
GR: Grisons
JU: Jura
LU: Lucerne
NE: Neuchâtel
NW: Nidwalden
OW: Obwalden
SG: St. Gallen
SH: Schaffhausen
SO: Solothurn
SZ: Schwyz
TG: Thurgau
TI: Ticino
UR: Uri
VD: Vaud
VS: Valais
ZG: Zug
ZH: Zurich

## 1 Background

Ensuring that hospital capacity meets population needs entails balancing quality, access, and costs of hospital care 1., 2., 3.. Economically viable and high-quality service delivery requires some degree of concentration [4], which can restrict access to hospital services [5]. Additionally, planning constraints vary with geographical, topographical, and demographic factors, as well as the nature and type of service. Successfully balancing quality, costs, and access is therefore complex and requires a sophisticated and targeted hospital planning approach 3., 6..

Internationally, hospital planning varies depending on national health objectives, political responsibilities, and regulatory frameworks 7., 8.. While several countries, such as Belgium, Finland, Germany, Luxembourg, Norway, Slovakia, and Switzerland, have recently reformed or are currently reforming their hospital planning 4., 9., 10., approaches differ depending on the health system's characteristics.

For example, a centralised approach to hospital planning can be observed in countries like Belgium, Denmark, France, Luxembourg, and Poland 1., 11., 12., 13.. Denmark's 2007 health system reform reduced the number of hospitals and focused on centralising specialised care. Evaluations indicate that the reform improved patient outcomes (e.g., mortality rates) and increased efficiency (e.g. shorter average hospital stays). The reform has maintained a high level of public trust. However, the restructuring of the hospital landscape led to slightly longer average travel times for patients [11]. Conversely, countries such as Germany, Italy, and Spain take a decentralised approach, with federal states or health regions being responsible for hospital planning 9., 14., 15.. In theory, decentralised health planning structures offer greater local authority and accountability, better addressing local healthcare needs [16].

In Switzerland's decentralised health system, the core building blocks of health policies and reforms are designed and legislated at the federal level. However, Switzerland's 26 self-governing cantons (legal administrative units that divide Switzerland into smaller spatial regions [17]) are responsible for the implementation of such reforms, including the concrete definition and enforcement of binding regulations 3., 17., 18.. Switzerland's hospital planning underwent reform with the revision of the Health Insurance Act (KVG) in 2007, and the Ordinance on Health Insurance (KVV) in 2009. The KVV is subordinate to the KVG and specifies and implements the KVG's provisions in detail [19]. The reform aimed to curb cost growth in the inpatient somatic care sector while maintaining access to high-quality care. The reform therefore introduced a Diagnosis-Related Group (DRG)-based payment system and free hospital choice for patients. At the same time, the reform specified cantonal hospital planning to steer health service provision. The reform required cantons to implement hospital planning between 2012 and 2014 and shifted from a resource- and capacity-oriented approach guided by the status quo to a service-, needs-, and quality-based approach [3]. Before 2012, cantons mostly planned their inpatient capacities based on beds. From 2012, cantonal hospital planning would be guided by three main principles: performance orientation, focusing on medical services; need-for-care orientation, ensuring that planning is based on need assessments and forecasting; and quality orientation, emphasising compliance with defined standards. Hospitals included on a canton's hospital list may bill treatments to compulsory health insurance and the patient's canton of residence, and this reimbursement is strictly limited to the service mandates granted. How to implement these three main principles in detail was up to the cantons [20]. Highly specialised medicine is not planned by the cantons but has been coordinated nationally since 2012 under the Inter-cantonal Agreement on Highly Specialised Medicine (IVHSM) [21].

The Canton of Zurich (ZH) played a leading role in developing a hospital planning approach that standardises the classification of medical services and links requirements to those services. Zurich therefore introduced hospital planning service areas (SPLB), subdivided into hospital planning service groups (SPLG), each defined by diagnosis and procedure codes 3., 22.. All services not assigned to one of these service groups fall into the basic package (Basispaket) for surgery and internal medicine, which every acute-care hospital on the hospital list must be able to provide. The basic package for elective surgery (Basispaket Chirurgie) is a reduced variant for hospitals without a comprehensive emergency service. The canton of Zurich then defined both general and service-specific requirements linked to the SPLG, including the availability of qualified specialists, defined levels of emergency and intensive care, minimum volume requirements, interdisciplinary cooperation, and regular tumour boards (for more details, see supplementary material 1). To receive the mandate of an SPLG, hospitals must meet the requirements 3., 23.. In the following chapters, we will refer to the operationalisation of the law and the ordinance of the canton of Zurich (as described above) as the SPLG method.

The 2022 amendment to the Ordinance on Health Insurance (KVV) further strengthened hospital planning by requiring cantons to disclose the factors used to forecast need for care and to assess economic efficiency and quality. Quality is assessed based on (non-binding) criteria, including qualified personnel, quality management systems, and minimum volume requirements (MVR). The KVV would, for example, say that for acute care hospitals, the cantons may impose MVR. It is then up to the cantons in their hospital planning to define if they use MVR and how high the thresholds are [19].

While the Zurich approach to hospital planning has been discussed before [3], gaps remain in understanding how the reform is implemented in the national context. While centralised health reform implementations, such as Denmark's 2007 health system reform [11], are well documented, decentralised systems present a more complex implementation landscape. Where subnational governments bear primary implementation responsibility, central authorities have limited tools to enforce uniform compliance, while subnational actors operate under distinct political incentives, administrative capacities, and local priorities. Implementation contexts vary across subnational governments, leading to implementation variation [24]. Despite recent and ongoing health system reforms in many countries, implementation lessons regarding reforms in decentralised health systems remain limited. Specifically, what factors influence variation in implementation across regions [4].

This study addresses this gap by examining how a federal hospital reform was implemented at the subnational level and why implementation may vary across subnational governments, using the Swiss reform as a case study and the Health Policy Triangle as an analytical framework 25., 26.. The framework distinguishes between the policy content, the actors involved, the process by which it is developed and implemented, and the context in which this takes place. It was chosen because it allows the interplay of actors, process and context to explain why initially identical policy content led to different outcomes in cantons with identical formal authority 25., 27..

The study aims to explain variation in implementation between Swiss cantons and, on that basis, identify which drivers of variation are likely to matter for other decentralised health systems undergoing similar reforms.

## 2 Methods

We followed a mixed-methods design combining document analysis of cantonal hospital planning materials, semi-structured expert interviews, and an exploratory quantitative analysis, with each component serving a distinct analytical purpose. The document analysis constituted the first step, systematically capturing observable variation across cantonal hospital lists and associated requirements, thereby establishing the empirical basis for subsequent analysis. Building on this foundation, the interviews were designed to access the contextual knowledge of key actors, to explore the reasons behind cantonal variation. The quantitative analysis then examined the extent to which observable structural cantonal characteristics were associated with deviations from the Zurich requirements, the baseline of our analysis. Together, these three components reflect the logic of methodological triangulation, with a dominant qualitative strand and a quantitative strand serving a confirmatory rather than a generative function.

### 2.1 Document analysis

The Swiss hospital planning outputs are cantonal hospital lists, i.e., tables that outline the approved service groups for every hospital. We conducted a document analysis [28] of all cantonal hospital lists available on cantonal websites, including all documents available through June 30, 2025. The aim was to identify differences in planning methods, service group definitions, and related general and service-group-specific requirements. Historical hospital lists dated back to 2012, while currently effective versions were published between 2015 and 2025. Further relevant materials, such as general and service-group-specific (quality) requirements, were reviewed (for detailed sources, see supplementary material 2).

Data from the documents were systematically compiled in a structured Excel file and analysed descriptively to allow inter-cantonal comparison. For each canton, we recorded (1) the year and version of the most recent hospital list and the planning interval; (2) the planning approach (SPLG method or other); (3) for cantons using the SPLG model, the Zurich version referenced and the number of defined service groups; and (4) the general and service-group-specific requirements attached to the service mandates.

To identify deviations, we compared each canton's requirements catalogue line by line with the Zurich catalogue of the version the canton references. A difference was coded as a deliberate deviation only if it was not attributable to the use of an older Zurich version. Within each dimension, deviations were classified as stricter (requirements added or thresholds raised relative to Zurich), less strict (requirements removed or thresholds lowered), or mixed (both within the same dimension, e.g. across service groups).

Based on the number of dimensions with deliberate deviations, cantons were grouped into three categories: no deviation (Zurich requirements adopted unchanged), deviation in a single dimension, and deviations in multiple dimensions.

### 2.2 Interviews

The study is reported in accordance with the Consolidated Criteria for Reporting Qualitative Research (COREQ) checklist (see supplementary material 3).

25 semi-structured interviews with cantonal representatives (*N* = 20) responsible for hospital planning in their department of health, and experts in Swiss hospital planning with a national or inter-cantonal perspective were conducted (*N* = 5, see supplementary material 4 for more information on the interviewees). The interviews complemented the document analysis, which shows what cantons decided but not why. The aim of the cantonal interviews was to gather more information on how the reform was implemented at the cantonal level and understand variation. The expert interviews addressed questions about the national dynamics of the reform and the dynamics between cantons (see supplementary materials 5 and 6 for the interview guides). All 26 cantonal representatives were invited to participate in the interviews via email. Of these, 20 cantons agreed to an interview. Six cantons declined an interview, primarily due to capacity constraints. One of these six cantons returned the interview guide with written answers, and the remaining five cantons provided only written information on the timeline for reform implementation. Additionally, two of the latter five cantons gave additional information in a 15-min call. Eight cantons provided additional documents on requirements that were not available as open access.

### 2.3 Data collection procedure

The interview guide was shared with participants before the interviews. Interviews were conducted between September 2024 and June 2025 by CW, CL and/or KL. Where necessary, interviews were supported by the individuals listed in the acknowledgements. Interviewees provided written consent acknowledging that their responses would be recorded, transcribed, and anonymised for research purposes. Interviews were conducted via Microsoft Teams or telephone, depending on participant preferences. Each session was audio-recorded. Interviews lasted between 45 and 60 min and were conducted in German (22/25) or French (3/25). Transcripts were not returned to participants for comment or correction, in line with the study design.

### 2.4 Data analysis

Each interview was transcribed verbatim. Following the framework approach to qualitative content analysis 29., 30., CL and CW first coded interview data using predefined deductive categories derived from the Health Policy Triangle (see supplementary material 7). To ensure that relevant aspects beyond the initial framework were not overlooked, open coding was applied in parallel, allowing inductive sub-codes to emerge directly from the data. All coded segments were subsequently organised in a thematic matrix, with individual interviews as rows and analytical categories as columns. This structure enabled systematic cross-case comparison across the 20 cantons interviewed; for the six remaining cantons, written information and planning documents were used. First-order codes were then iteratively abstracted into higher-order themes reported in this paper.

### 2.5 Exploratory cluster analysis

To complement the qualitative findings, we conducted an exploratory, unsupervised cluster analysis. This approach is used to discover patterns in the data that indicate group boundaries and thereby enable grouping of cases [31]. We examined whether structural cantonal characteristics were associated with the degree of deviation from the Zurich requirements. The dataset included variables such as the degree of urbanisation; age distribution; hospitalisation rate per 1000 inhabitants; and major region assignment. All continuous variables were z-standardised, while the major region variables were retained in their original form. All variables are taken from the cantons' most recent planning year (see supplementary material 8).

Using the standardised data, we applied a k-means clustering algorithm to identify latent groups of cantons. The optimal number of clusters (k = 3) was determined using the Elbow method [32]. Each canton was assigned to a cluster, and the clusters were characterised by the input variables across groups. We then examined whether cantons within the same cluster showed comparable deviation in their requirements, using the three deviation categories derived from the document analysis. All analyses were performed using the kmeans() function from the base R package *stats* in R (version 4.5.2). The resulting clusters were subsequently compared with the categorisation derived from the document analysis to explore potential associations.

## 3 Results

The results are organised according to the four dimensions of the Health Policy Triangle, examining how implementation unfolded, what was implemented, the context shaping implementation, and the actors who influenced reform implementation across cantons.

### 3.1 Process: How implementation unfolded

Fig. 1 shows when each canton implemented the part of the national reform that encompasses hospital planning (blue dots) and when the cantons implemented the SPLG method (green dots).

**Fig. 1:**
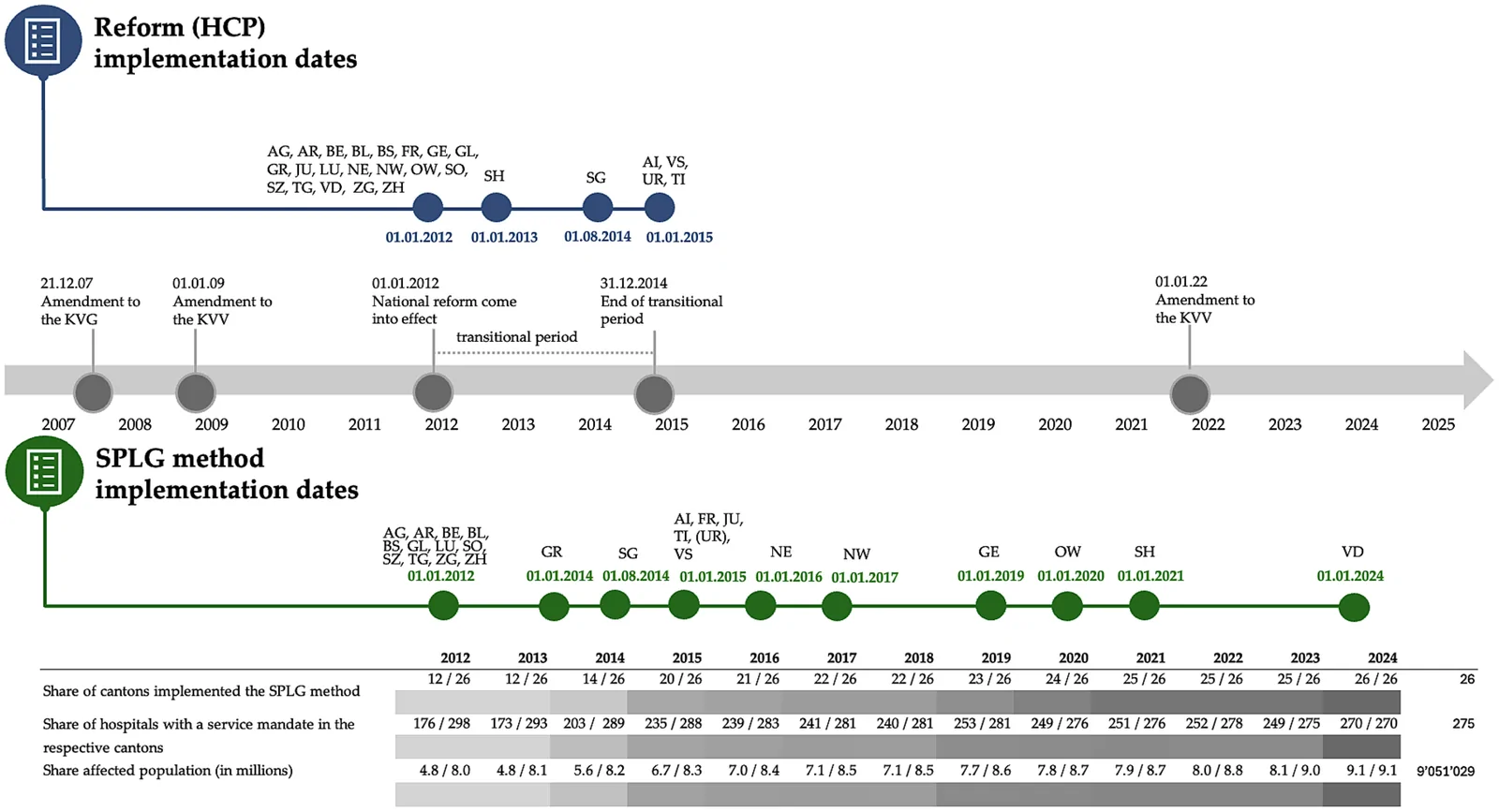
Timeline of the KVG reform and SPLG implementation across cantons.

Annotation: Abbreviations of the cantons can be found in the glossary. Grey dots indicate the national amendments to the KVG and KVV related to HP, blue dots indicate the KVG implementation (HP) dates, green dots indicate the SPLG method implementation dates across cantons. Uri (UR) is in brackets, as UR only implemented SPLB. The information on implementation dates comes from the interviews. The grey bars reflect the share of how many cantons have adopted the SPLG method and, conversely, how many hospitals and how many people were affected 33., 34..

Twelve cantons implemented the SPLG method alongside Zurich in 2012, while others followed in subsequent years, with Vaud (VD) being the last to adopt it in 2024. The canton of Uri (UR) is an exception, as it only assigns mandates at the SPLB level. In general, French-speaking cantons adopted the model later than German- and Italian-speaking regions.

### 3.2 Content: Convergence in SPLG definitions, divergence in minimum volume requirements

While the policy content of the KVG/KVV revision is identical for all cantons, the content of the SPLG model as applied differs between cantons.

### 3.3 Differences in the SPLG definitions

The SPLG outline the specific medical treatments and procedures that belong to each service group [3]. The first version of Zurich (version 2012) had 25 SPLB and 143 SPLG, including four SPLG that are part of the IVHSM. More than a decade later, the hospital list (version 2025.1) still includes the same 25 areas, but now covers 161 SPLG, including 35 SPLG for IVHSM (see supplementary material 9).

When it comes to the definition of SPLG and SPLB, cantonal approaches vary (see Table 1). While for some cantons variations come from different versions of the SPLG method, other cantons do not consider certain SPLG and/or define additional SPLG. Most differences can be observed in the SPLB basic packages and in the interdisciplinary areas, such as geriatrics. Valais (VS), for instance, a geographically large, bilingual, and mountainous canton, assigns service mandates for 134 SPLG, whereas 20 SPLG are part of IVHSM. Out of the 134, VS defines 15 SPLG that are mandated only to university hospitals outside the canton, but are not part of the IVHSM, such as intestinal and spleen transplants (TPL 6 and 7). The cantons of St. Gallen (SG), Appenzell Inner-Rhodes (AI) and Appenzell Outer-Rhodes (AR), which published a joint hospital list in 2024, created an additional SPLG in the SPLB basic packages called “Health and emergency centres” (GNZ). This SPLG was created to enable outpatient emergency centres at former hospital locations to operate short-stay emergency beds [35]. Other cantons defined adaptations as well: For example, Fribourg (FR) assigns service mandates for the SPLG “crisis unit”, while Uri (UR), a mountainous canton with a low population density and with one hospital, does not assign service mandates for SPLG like “Birth centres” (GEBH).

**Table 1:** Cantonal characteristics and planning particularities.

Canton	Urbanisation (%, 2024)	Population density (2024)	Hospital density (2023)	Bed density (2023)	Last conducted HCP in the canton	HCP cycle (years)	Last hospital list	Number of hospitals in the canton with service mandate	Number of SPLG
AG	62.8	518	1.51	1.94	2024	4	2025	11	126
AI	72.9	96	0	0.00	2023	10	2024	0	127
AR	28.1	233	5.31	3.04	2023	10	2024	3	127
BL	69.1	577	3.68	1.92	2021	4	2025	11	122
BS	100	5.406	5.00	7.03	2021	4	2025	10	122
BE	53.3	178	2.54	2.57	2019	10	2025	27	122
FR	32.4	204	1.46	1.71	2015	10	2015	5	121
GE	95.1	1.858	1.72	3.10	2025	Not fixed	2025	9	126
GL	77.2	61	2.38	1.62	2015	Not fixed	2015	1	121
GR	32.9	29	7.32	2.76	2014	10	2022	15	120
JU	33.7	89	4.02	2.21	2023	8–10	2023	3	126
LU	54.8	290	2.08	2.30	2016	6	2024	9	120
NE	64.8	222	1.68	2.19	2016	7	2016	3	120
NW	36.5	163	4.44	1.58	2017	10–15	2017	2	119
OW	27.4	80	2.55	1.30	2020	Not fixed	2024	1	120
SH	57.3	292	2.30	2.17	2025	10	2025	2	122
SZ	69	184	2.39	1.45	2015	10	2025	4	117
SO	60.4	363	1.05	1.78	2017	10	2024	3	122
SG	68.3	264	2.43	2.51	2023	10	2024	13	127
TG	45.7	298	1.35	1.83	2023	10	2023	4	126
TI	73.2	127	3.63	3.62	2023	10	2023	13	126
UR	62	35	2.64	1.50	2015	10	2023	1	–
VS	55.1	70	2.19	1.96	2015	10	2023	8	114
VD	63.1	263	3.31	2.80	2024	10	2024	28	125
ZG	91.6	555	2.26	1.59	2023	10	2024	3	115
ZH	79.3	929	1.56	2.42	2023	10	2025	25	126

Annotation: For the table, the latest available data was used until June 30, 2025. The degree of urbanisation indicates the proportion of the permanent resident population living in urban municipalities [34]. Urban municipalities are defined according to the federal government's spatial typologies [36]. The population density is calculated as inhabitants per sq. km in 2024 [34]. Hospital density is calculated as hospitals in the canton per 100,000 inhabitants in 2023 [37]. Bed density is calculated with the number of beds per 1000 inhabitants in 2023 [37]. The number of beds per canton excludes beds in psychiatry and rehabilitation. The HCP cycle indicates the intervals in which the canton conducts hospital capacity planning. The sources to the latest hospital lists and the number of SPLG can be found in supplementary material 2.

Other differences from the most current Zurich planning version are due to cantons using older Zurich planning versions. For example, Zurich's 2018 revision split “Reconstruction of the Lower Extremities” (BEW7) into “Primary Knee Replacement” (BEW 7.2) and “Revision Knee Arthroplasty” (BEW 7.2.1), yet some cantons still group all hip and knee replacements and revisions in the one BEW7 SPLG.

### 3.4 Differences in quality requirements

To receive a service mandate, hospitals must comply with predefined general and SPLG-specific requirements. The definition and application of SPLG-specific requirements vary across cantons, primarily for two reasons. First, fifteen cantons, such as Glarus (GL), Ticino (TI) and VS, base their requirement specifications on earlier versions of the Zurich requirements. Second, cantons make deliberate deviations from the requirements.

### 3.5 Variation due to older versions

Certain requirements have changed over time, which leads to differences among cantons that rely on older versions of Zurich's planning documents. For example, tumour board requirements deviate in 13 cantons from the ZH2025 version because these cantons still use earlier versions that include other specifications, such as the required frequency of boards. Variation stems from differing planning cycles (see Table 1), which determine how often cantons revise their hospital planning and hospital lists.

### 3.6 Variation due to deliberate deviations

Several cantons adopted the SPLG method but deliberately modified specific requirements of the Zurich version that served as their template (Table 2). These deviations are deliberate choices in hospital planning and are distinct from differences that arise merely from using an older Zurich version.

**Table 2:** Differences in quality requirements across cantons.

Canton	SPLG requirements in use	Count of deviations	Requirements are stricter	Requirements are less strict	Mixed
AG	ZH 2023	9 of 10	Specialist qualifications; Tumour board	Basic packages; MVR	Specialist availability; Emergency; ICU; Cooperation (both)
BE	ZH 2025	7 out of 10	Specialist qualifications; Tumour board	MVR	Specialist availability; Emergency; ICU; Cooperation (ext.)
AI	ZH 2023	6 out of 10	Specialist availability; Emergency; Cooperation (both); Tumour board		MVR
AR					
SG					
TG	ZH 2023	3 out of 10	Emergency; Tumour board	MVR	
GR	ZH 2016	1 out of 10		MVR	
LU	ZH 2019	1 out of 10		MVR	
NW	n.a.	1 out of 10		MVR	
OW	n.a.	1 out of 10		MVR	
ZG	ZH 2020	1 out of 10		MVR	

Annotations: The canton of Zurich (ZH) serves as the baseline; only cantons that deliberately deviated from the service-group-specific requirements of the ZH version they adopted are shown. “Stricter” indicates that the canton introduced requirements that go beyond those of ZH (additional requirements or higher thresholds); “less strict” indicates requirements below those of ZH (requirements removed or thresholds lowered); “mixed” indicates that, within the same requirement dimension, some requirements are stricter and others less strict than in ZH. The count refers to the number of requirement dimensions (out of ten) in which a canton deviates. Information on minimum volume requirements (MVR) was taken from the cantonal requirement documents and, where necessary, verified in the interviews.

Regarding emergency care levels, Thurgau, for example, differentiates between internal and external emergency services, while most other cantons adopt four levels of emergency care (internal only). All hospitals in the canton of Thurgau must cooperate with the two cantonal hospital sites to coordinate emergency care with the goal of increasing the efficiency of emergency care provision. Aargau (AG) and Bern (BE) show the greatest deviations from Zurich's requirements. One interviewee described the deliberate deviations as follows: *The Bern model allows more flexibility, especially for emergency departments. Zurich is relatively inflexible, whereas Bern theoretically allows emergency department duties to be temporarily delegated to other hospitals, for example, during the night. Interestingly, in Bern this mainly applies to the urban hospitals, and we would need this exception for regional hospitals, that is, for rural care.*

Minimum volume requirements (MVR) are most often adapted by cantons. Zurich introduced MVR per hospital in 2012 and per surgeon in 2019 [38]. However, not all cantons have adopted them, as outlined in Fig. 2.

**Fig. 2:**
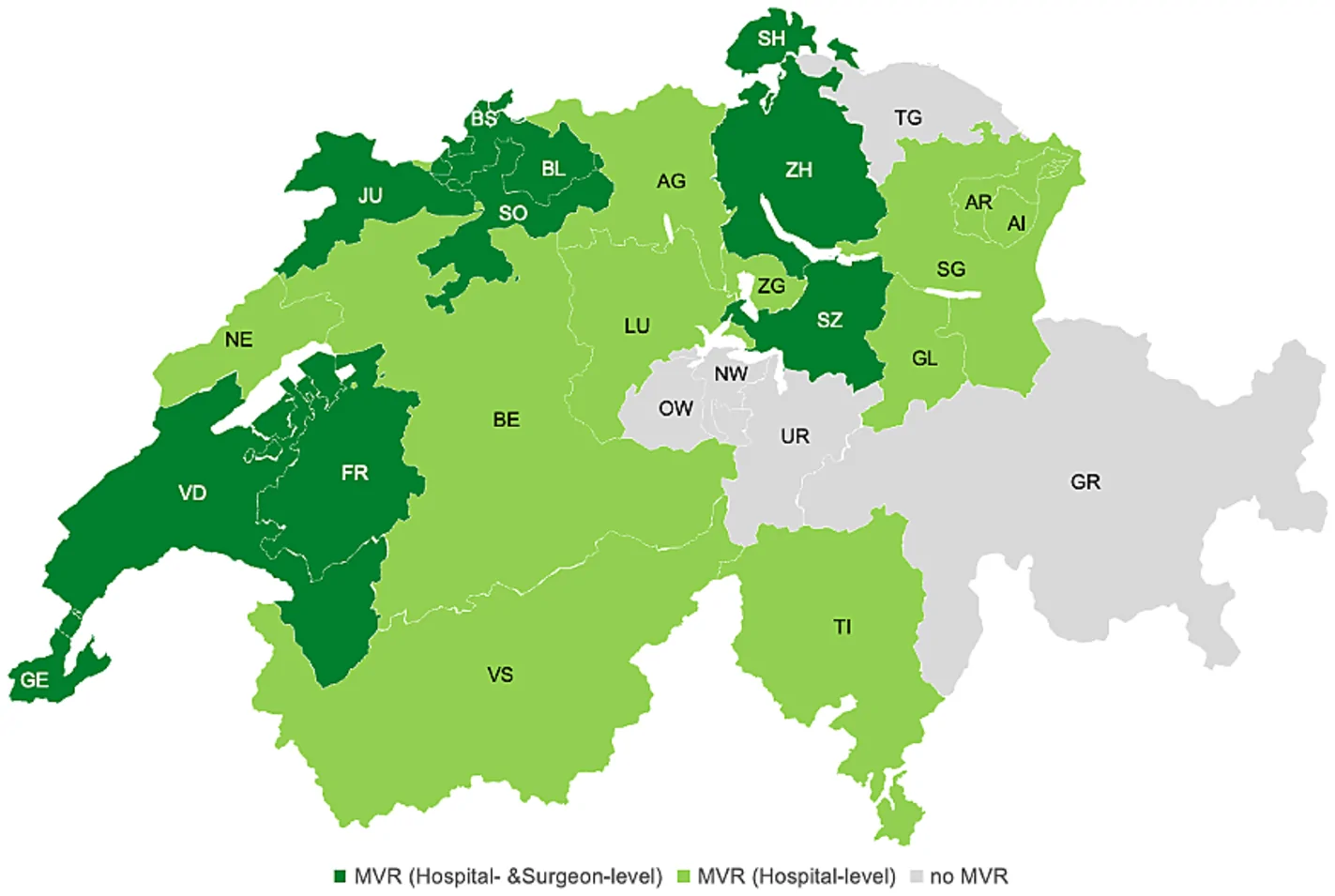
Adoption of minimum volume requirements at hospital and surgeon levels across cantons. Annotations: Abbreviations of the cantons can be found in the glossary. Dark green cantons have implemented MVR at hospital- and surgeon levels, while light green cantons have implemented MVR only at hospital level. Grey colour-coded cantons do not yet work with MVR (as of June 2025). The information on MVR comes from the cantonal requirement documents and/or were verified during the interviews.

Among Switzerland's 26 cantons, ten have introduced MVR specifying thresholds for individual surgeons and hospitals, while eleven apply them only at the hospital level. Five cantons do not apply MVR. Across cantons using MVR, thresholds are defined for 33 SPGLs, with some variation. For example, the thresholds for initial hip replacement (BEW7.1) differ for Zurich (50 for hospitals, 15 for surgeons) and Bern (20 for hospitals), as well as for spine surgery (BEW8), where the threshold is 100 for Zurich hospitals and 10 for Bern hospitals.

### 3.7 Context: three themes shaping implementation variation

#### 3.7.1 Administrative capacity as a driver of variation

While all cantons acknowledged and appreciated Zurich's pioneering role in developing the SPLG method, they emphasised the significant differences between Zurich and their own contexts, particularly regarding resources available for hospital planning. Limited administrative resources can deter cantons from revising their planning more frequently, as expert 1 highlighted: *Zurich has an entire department dedicated to this, with people who do the controlling and others who analyse the data. In other cantons, one person has to do everything, and the resources available are completely different.* Especially small cantons have flagged their administrative resources: *We previously had a hospital list that was effective indefinitely, because hospital planning requires a great deal of resources. And we are a relatively small canton with limited administrative resources.* This lack of internal capacity is one reason why smaller cantons have expressed a desire for increased collaboration across regions. As of June 2025, two inter-cantonal collaborations are active: one between Basel-Stadt and Basel-Land (BS/BL) and another among St. Gallen, Appenzell Inner-Rhodes and Appenzell Outer-Rhodes (SG/AI/AR). Thurgau (TG) will join the SG/AI/AR collaboration for 2031 [39].

#### 3.7.2 Linguistic and cultural context leading to initial resistance

The cultural divide between French- and German-speaking cantons was a recurring topic in the interviews. One French-speaking canton reported that although it relies on Zurich's hospital planning documents, it avoids publicly referencing them due to concerns about public or political resistance.

#### 3.7.3 Different local characteristics of the cantons

The characteristics of cantons differ, as seen in Table 1. Cantons highlight, that they have different regional circumstances compared to Zurich. These circumstances were named as drivers for variation, especially for rural cantons. As an example, cantons adjust specialist qualification requirements when several hospitals have difficulties meeting them: *What we do on a case-by-case basis is that when we see that several hospitals are having problems meeting certain requirements, we discuss it with the hospitals to see if there is an alternative. For example, if Zurich, a city canton, says that acute geriatrics requires a geriatrician, and we then notice that our hospitals do not have that specialist, we adjust the requirements for that service mandate, as long as the quality is equivalent.* Furthermore, when it comes to the requirements, individual cantonal deviations are made, as this cantonal representative stated: *While we adopt the systematic and therefore also the requirements in principle, we can deviate from a one-to-one application in individual cases if this is indicated for care purposes due to regional circumstances. To my knowledge, most cantons make such individual deviations.*

The same holds true for MVR. MVR enforcement varies, and when hospitals cannot meet them, MVRs are often relaxed. A cantonal representative highlighted: *We have deviated somewhat from the minimum volume requirements in some cases because we realised that we cannot quite meet them for individual services, as we do not have as many cases in the entire region as are required.* For obstetrics (GEB 1 and GEB1.1), all cantons with MVR set a target of 1500 cases for the two SPLG, except BE, which set a target of 1000. The interviewee from a rural canton with dispersed settlements across mountainous terrain, where MVR were not introduced, explained: *In the canton, we are talking about just over 1000 births per year, which would mean only a single maternity ward. However, even by private car, travel times from a remote valley can exceed two hours.*

Finally, to examine whether structurally similar cantons implemented comparable deviations from the Zurich SPLG model, we grouped cantons based on the variables defined in the methods section. The Elbow Method determined three clusters. Cluster 1 comprises high-alpine, sparsely populated cantons with substantial elevation differences and slightly above-average hospitalisation rates and length of stay. Cluster 2 includes highly urban, densely populated cantons with minimal topographic variation and the longest average length of stay. Cluster 3 contains all remaining cantons, characterised by near-average values across most variables. However, when linking these clusters to deviation from the Zurich requirements, no systematic pattern emerged. These results indicate that **structural characteristics that are straightforward to observe alone cannot explain the degree of** deviation **from the** Zurich **requirements (see** supplementary **material 10).**

### 3.8 Actors: Zurich setting the standard, cantonal health directorates under political pressure

Two actor dynamics shaped implementation. First, the Zurich model diffused voluntarily across cantons. It was never legally mandated at the national level, yet it became the de facto standard for Swiss hospital planning. This diffusion was driven by Zurich's technical credibility and the absence of comparable alternatives. Most interviewed cantons voluntarily noted that the development of the SPLG by the canton of Zurich led to the professionalisation of hospital planning. As expert 2 elaborated: *I think the biggest influence is that the SPLG have been defined throughout Switzerland, and everyone is talking about the same thing.* Most cantons acknowledged Zurich's pioneering role, underscoring that convergence in this case emerged bottom-up through voluntary adoption rather than top-down through legal enforcement. Second, the health directorates faced political pressure, especially for the enforcement of MVR. One **inter-cantonal collaboration** failed because cantons could not agree on MVR: *The cantons […] have not used minimum volume requirements to date, and they were not prepared to use them. For us, this was a non-negotiable condition.* Expert 1 noted that leniency is driven more by political factors than by medical necessity. One cantonal representative highlighted, that not granting certain service mandates are very sensitive for politicians, as it can lead to the destabilisation of local hospitals: *We know that many health directors run the risk of being voted out of office if they close hospitals.* Another cantonal representative noted: *A hospital has voter potential, as it is often a major employer in the region or for a canton, and that creates a certain amount of political pressure.* Despite tensions around MVR, 18/22 cantons and all experts agreed that there is a relationship between volume and outcome.

## 4 Discussion

This study examined how a federal hospital planning reform was implemented in a decentralised health system using Switzerland as a case study. We analysed how and why implementation varied across subnational governments and explored whether deviations might be connected to structural characteristics such as population density or settlement structure. Applying the Health Policy Triangle [26], our findings show that policy content, implementation context, process, and actors shaped implementation variation. Convergence and divergence emerged through mechanisms with broader implications for decentralised health systems, as outlined in Fig. 3.

**Fig. 3:**
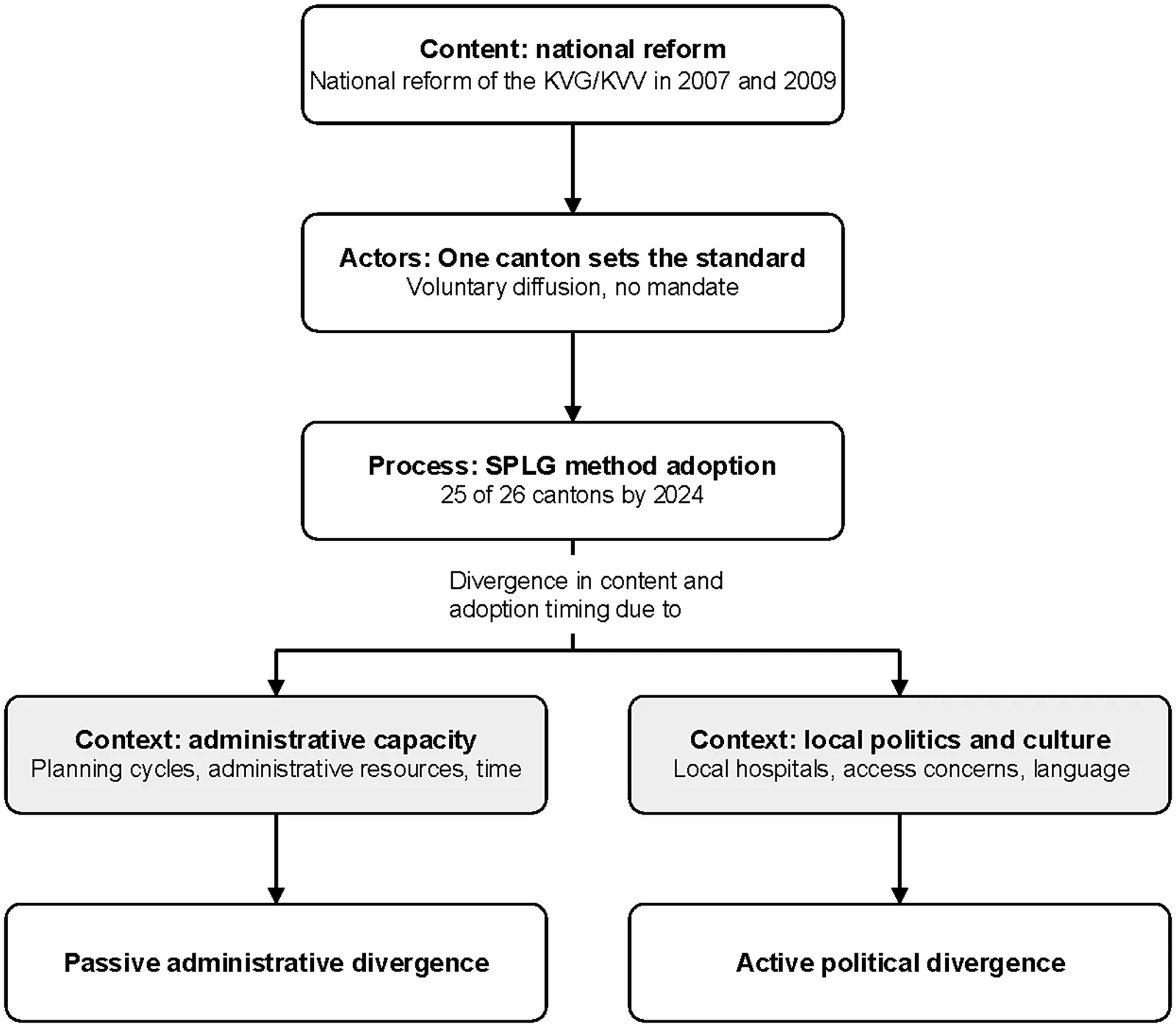
Interplay of policy content, implementation context, process, and actors in the cantonal implementation of the hospital planning reform.

While cantons were autonomous in implementing the national reform, the content dimension reveals diffusion toward a common planning methodology. The actor dimension highlighted Zurich's role, which demonstrates that voluntary diffusion of subnational policy innovations can produce quasi-national standards. In federal states, policy diffusion is a common phenomenon, allowing the development and spread of best practices, as discussed in [40].

By 2024, 25 cantons had adopted the SPLG method, while one canton only adopted SPLB. Adoption timing is linked to linguistic boundaries, cantonal planning cycles and administrative resources. More significantly, the process dimension shows inter-cantonal collaboration emerging in response to capacity constraints.

The context dimension provides further explanations for observed variation. Differences in SPLG definitions largely reflect administrative capacity and different planning cycles rather than disagreement over the content. Deviations in the number and definition of SPLGs occurred only in single instances, mainly to accommodate cantonal hospital-landscape particularities, such as former hospitals now operating as health centres. Interestingly, we observe no deviations in French-speaking cantons. The cultural reluctance identified in the interviews regarding implementation timing does not translate into reluctance regarding content.

The greatest variation emerged in MVR. Cantons implemented MVR at the hospital and/or surgeon level, and thresholds differed for certain SPLGs. These deviations were not driven by doubts about the volume-outcome relationship, but by concerns regarding access to care in mountainous regions, political feasibility, and the risk of destabilising local hospitals. Interviewees emphasised that hospitals are politically sensitive institutions. Closing or downsizing them may have electoral consequences. This concern is not unfounded. The literature shows that hospital closures in rural areas reduce local welfare and impact voting behaviour 41., 42.. The exploratory clustering analysis further indicated that structurally similar cantons made deviations from the original Zurich requirements to varying degrees. This supports the qualitative finding that deviations are likely to be shaped by political incentives and administrative capacity. This is consistent with Campos and Reich's framework of political analysis for health policy implementation, which emphasises that implementation gaps are often not technical but political [24]. This raises concerns about whether such flexibility is applied proportionately to balance access, costs and quality, or whether it primarily serves to avoid politically sensitive decisions. The findings mirror what the literature describes as **vertical fragmentation in federal systems**. In decentralised governance structures, central authorities have limited tools to enforce uniform implementation, while subnational units make political decisions [24].

The linguistic and cultural divide between French- and German-speaking cantons represents another contextual factor that influenced adoption timing. The finding that one French-speaking canton avoids publicly referencing Zurich documents despite relying on them is an illustration of how, in federal health systems, subnational identity intersects with policy adoption [43].

Administrative capacity represents another contextual factor. For smaller cantons, limited hospital planning resources produce passive divergence, which reflects inertia rather than choice. This distinction between passive divergence and active adaptation is important and has practical implications. Shared technical resources and inter-cantonal collaboration may reduce passive divergence, whereas active deviation is politically motivated.

### 4.1 Implications for policy and research

Three findings, in our view, are likely to apply beyond Switzerland. First, the distinction between passive and active divergence matters, as contexts differ across regions. Second, a well-resourced region can develop a method in response to a reform that can diffuse into a quasi-national standard without any legal mandate. This depends only on asymmetric capacity and regional discretion, both common in decentralised systems. Third, deviations concentrate on politically sensitive requirements, above all MVR, rather than on service classification, because the former have visible consequences for local hospitals. By contrast, the timing of adoption, the role of language regions, and the influence of direct democracy reflect Swiss institutional features and should be read as case-specific.

The challenge for federal systems is designing mechanisms that preserve local responsiveness while ensuring that deviations are guided by considerations of costs, quality and access rather than political considerations. Therefore, we propose the following policy recommendations for Switzerland and beyond:

1. Strengthen national standardisation. A central institution should develop and maintain standards such as definitions of service group and quality requirements. Centralising the technical standard-setting process can furthermore reduce the political bias of cantonal decisions, decrease administrative effort (particularly for small cantons) and support inter-cantonal planning.
2. Allow regional flexibility while ensuring transparency. To maintain responsiveness to local needs, cantons should retain flexibility to adapt certain planning requirements to local circumstances. Transparent deviation rules, within a defined range and for specific requirements, would help ensure accountability and limit purely politically motivated exceptions. Deviations can be accompanied by systematic impact assessments to evaluate their effects on access, quality, and costs, enabling learning and adjustment over time.

International comparisons show similar efforts to balance national standards with local flexibility. Italy's hospital planning is decentralised, but national guidance defines standards for bed numbers, hospital classification, and MVR, while regions adapt implementation to local conditions [44]. Germany has recently adopted a comparable approach with its 2024 hospital reform, introducing nationally defined service groups and quality requirements, while hospital planning remains with the federal states (Bundesländer), reflecting a federal structure similar to Switzerland 9., 45.. Ongoing political debates in Germany focus on whether explicit exceptions to national standards should be defined, as reflected in the draft “Hospital Reform Adjustment Act” (KHAG) [46].

### 4.2 Limitations and future research

We highlight five main limitations. First, the document analysis captures intended rather than actual practice. Published requirements may be enforced loosely and negotiated differently with individual hospitals. We reduced this risk by verifying deviations in interviews, but it remains theoretical, as verifying actual practice is beyond this study's scope. Second, the qualitative data rely on self-reported information by the cantonal representatives who were sometimes involved in the decisions studied. Accounts of why a canton adopted or modified the SPLG model may reflect recall bias and, particularly for sensitive requirements such as MVR, political framing. Triangulation with documents and with the expert interviews limits but does not remove this risk. Third, the cluster analysis is descriptive and does not allow causal interpretation; it was designed to test whether observable structural conditions could account for deviation patterns, not to generate a typology of cantons. A design in which the classification precedes the qualitative strand, using cluster membership to derive hypotheses that are then probed with actors, would allow structural configurations to be interrogated directly and is a promising avenue for future comparative work.

Fourth, the Health Policy Triangle is a generic, descriptive framework. It allowed us to examine the interplay of content, actors, process and context. Supplementing the analysis with additional frameworks such as the advocacy coalition framework or the Kingdon's multiple stream theory model would enrich the analysis and further explain our findings [25].

Finally, institutional features specific to Switzerland shape the extent of divergence. Cantons act as planners, co-financiers and, often, owners of hospitals, leading to different and sometimes opposing incentives. Lastly, since the study's data collection ended in 2025, we have observed further convergence among the cantons, even on politically sensitive requirements. Further research is needed to better understand convergence on highly political dimensions.

Future quantitative research should examine how differences in requirements affect hospital quality, costs, and service centralisation.

## 5 Conclusions

This study examined how a national health reform was implemented at the subnational level in Switzerland and how and why implementation varied across cantons. Despite autonomous implementation responsibility, cantons have largely converged on a common planning method. These findings suggest that alignment in hospital reform implementation can emerge within decentralised health systems. Yet, meaningful variation persists. The study observed passive divergence driven by differences in administrative capacity, and active divergence driven by politically sensitive decisions. National standard-setting could reduce passive divergence, while transparent regional flexibility could channel active divergence into accountable variation.

## Declaration of generative AI in scientific writing

During the preparation and revision of this work, the authors used ChatGPT, Grammarly and Claude.AI to improve the language, readability, and clarity of specific paragraphs. After using these tools, the authors reviewed and revised the text and take full responsibility for the content of the published article.

## CRediT authorship contribution statement

**Carla Walker:** Writing – original draft, Visualization, Software, Project administration, Methodology, Formal analysis, Data curation, Conceptualization. **Charlotte Lang:** Writing – original draft, Visualization, Methodology, Formal analysis, Data curation, Conceptualization. **Daria Bukanova-Berend:** Writing – review & editing, Data curation. **Katharina Lonnes:** Writing – review & editing, Data curation. **David Ehlig:** Writing – review & editing, Methodology, Conceptualization. **Justus Vogel:** Writing – review & editing, Methodology, Conceptualization. **Dr. Alexander Geissler:** Writing – review & editing, Supervision, Methodology, Funding acquisition, Conceptualization.

## Consent for publication

Each interview participant signed a consent for publication.

## Ethics approval and consent to participate

This study does not fall within the scope of the Swiss Federal Act on Research involving Human Beings (Human Research Act, HFG), as it does not constitute research on human diseases or the structure and function of the human body. Therefore, approval by a cantonal ethics committee was not required.

All interview participants were informed about the purpose of the study, the recording of the interviews, and the use of the data for research purposes. Written informed consent was obtained from all participants prior to participation.

## Funding

This research was supported by the Swiss National Science Foundation (SNSF) (grant number: 10001646).

## Declaration of competing interest

The authors declare the following financial interests/personal relationships which may be considered as potential competing interests: Prof. Dr. Alexander Geissler reports financial support was provided by Swiss National Science Foundation. Charlotte Lang, Dr. David Ehlig, Dr. Justus Vogel and Prof. Dr. Alexander Geissler reports a relationship with Groupe Mutuel Foundation that includes: consulting or advisory. Dr. David Ehlig, Dr. Justus Vogel and Prof. Dr. Alexander Geissler reports a relationship with Health Departments of the Cantons of St. Gallen, Appenzell Ausserrhoden, and Appenzell Innerrhoden that includes: consulting or advisory. Charlotte Lang, Daria Bukanova-Berend, Dr. David Ehlig, Dr. Justus Vogel and Prof. Dr. Alexander Geissler reports a relationship with OECD that includes: consulting or advisory. Carla Walker, Charlotte Lang, Dr. David Ehlig, Dr. Justus Vogel and Prof. Dr. Alexander Geissler reports a relationship with Canton of Schwyz that includes: consulting or advisory. Carla Walker reports a relationship with walkerproject ag that includes: employment. If there are other authors, they declare that they have no known competing financial interests or personal relationships that could have appeared to influence the work reported in this paper.

## Data Availability

Stakeholder interview data generated during the current study are not publicly available due to the lack of participant consent for data sharing but are available anonymized from the corresponding author on reasonable request. Hospital lists as well as general and service-specific requirements were obtained from publicly available cantonal websites and through direct e-mail requests. Anonymised copies of the compiled dataset are available from the corresponding author on reasonable request. All data underlying the exploratory clustering analysis are included in this published article and its supplementary information files. The R code used for the exploratory clustering analysis is available from the corresponding author upon reasonable request.

## References

[1] Dubas-Jakóbczyk K., Sowada C., Domagała A., Więckowska B. (2018). Building hospital capacity planning mechanisms in Poland: the impact of 2016/2017 regulatory changes. Health Planning & Management.

[2] Rechel B., Wright S., Barlow J., McKee M. (2010). Hospital capacity planning: from measuring stocks to modelling flows. Bull World Health Organ.

[3] Bleibtreu E., Von Ahlen C., Geissler A. (2022). Service-, needs-, and quality-based hospital capacity planning – the evolution of a revolution in Switzerland. Health Policy.

[4] Polin K., Hjortland M., Maresso A., Van Ginneken E., Busse R., Quentin W. (2021). “Top-three” health reforms in 31 high-income countries in 2018 and 2019: an expert informed overview. Health Policy.

[5] Avdic D. (2016). Improving efficiency or impairing access? Health care consolidation and quality of care: evidence from emergency hospital closures in Sweden. J Health Econ.

[6] Ettelt S., Nolte E., Thomson S., Mays N., Network I.H.C. (2008).

[7] Rechel B. (2009).

[8] Geissler A., Busse R. (2015).

[9] Subelack J., Kuklinski D., Vogel J., Geissler A. (2024).

[10] Rausch K., Durvy B., Goetzinger C., Winkelmann J., Lorcy A.-C., Falkenbach M. (2025).

[11] Christiansen T., Vrangbæk K. (2018). Hospital centralization and performance in Denmark—ten years on. Health Policy.

[12] Fornaciari D., Vleugels A., Callens S., Eeckloo K. (2011). Monitoring quality in a Federal State with shared powers in healthcare: the case of Belgium. Eur J Health Law.

[13] Chrusciel J., Clément M.-C., Steunou S., Prost T., Duclos A., Sanchez S. (2023). Effect of the implementation of the French hospital regionalization policy on patient mobility. Health Syst Reform.

[14] Belvis AG de, Meregaglia M, Morsella A, Adduci A, Perilli A, Cascini F, et al. Italy: health system review. Health Syst Transit 2022;24:1–236. 36951263

[15] Bernal-Delgado E., Angulo-Pueyo E., Ridao-López M., Urbanos-Garrido R.M., Oliva-Moreno J., García-Abiétar D. (2024). Spain: health system review. Health Syst Transit.

[16] Windle A., Javanparast S., Freeman T., Baum F. (2025). Use of evidence to inform regional primary health care planning in Australia. Health Res Policy Sys.

[17] (2024). Political system About Switzerland.

[18] Spycher J., Morisod K., Moschetti K., Le Pogam M.-A., Peytremann-Bridevaux I., Bodenmann P. (2024). Potentially avoidable hospitalizations and socioeconomic status in Switzerland: a small area-level analysis. Health Policy.

[19] Bundesrat Der Schweizerische (2025). Verordnung über die Krankenversicherung (KVV). Stand am.

[20] Lobsiger M., Frey M. (2019). Evaluation der KVG-Revision im Bereich der Spitalfinanzierung. Auswirkungen der Revision auf die Kosten und die Finanzierung des Versorgungssystems. Schlussbericht Bundesamt für Gesundheit BAG.

[21] Moser D, Ahlen C von, Geissler A. Qualitätsvariation in der Schweiz: Analyse der Mamma-Resektionen bei Brustkrebs anhand des Qualitätsindikators der Brusterhaltung. Das Gesundheitswesen 2022;84:539–46. doi:10.1055/a-1670-7249. PMC11248625 34847592

[22] https://www.zh.ch/de/gesundheit/spitaeler-kliniken/spital-leistungsgruppen-und-splg-grouper.html.

[23] (2025). Gesundheitsdirektion Kanton Zürich. Anhang zur Zürcher Spitalliste 2023 Akutsomatik: Leistungsspezifische Anforderungen (Version 2025.1; gültig ab 1. Januar 2025).

[24] Campos P.A., Reich M.R. (2019). Political analysis for health policy implementation. Health Syst Reform.

[25] O'Brien G.L., Sinnott S.-J., Walshe V., Mulcahy M., Byrne S. (2020). Health policy triangle framework: narrative review of the recent literature. Health Policy OPEN.

[26] Walt G., Gilson L. (1994). Reforming the health sector in developing countries: the central role of policy analysis. Health Policy Plan.

[27] Peckham S. (2023). Is it time to explore the health policy process within governance and health systems frameworks? Comment on “modelling the health policy process: one size fits all or horses for courses?”. Int J Health Policy Manag.

[28] Bowen G.A. (2009). Document analysis as a qualitative research method. Qual Res J.

[29] Gale N.K., Heath G., Cameron E., Rashid S., Redwood S. (2013). Using the framework method for the analysis of qualitative data in multi-disciplinary health research. BMC Med Res Methodol.

[30] Ritchie J., Spencer L., Bryman A., Burgess R.G. (1994). Analyzing qualitative data.

[31] Milligan G.W., Cooper M.C. (1985). An examination of procedures for determining the number of clusters in a data set. Psychometrika.

[32] Kodinariya Trupti, Makwana Prashant (2013). Review on determining number of cluster in K-means clustering. International Journal of Advance Research in Computer Science and Management Studies.

[33] SwissDRG A.G. (2026). Liste der Netzwerkspitäler 2012–2024. SwissDRG.

[34] (2025). Bundesamt für Statistik (BFS). Struktur der ständigen Wohnbevölkerung nach Kanton, 2010–2024.

[35] Unternährer Appenzeller R., Prey H., Kuklinski D., Vogel J. (2024).

[36] (2025). Major regions.

[37] Bundesamt für Gesundheit (BAG) (2023). Kennzahlen der Schweizer Spitäler.

[38] Vogel J., Polin K., Pross C., Geissler A. (2019).

[39] Kanton Thurgau (2025). Ostschweizer Kantone einigen sich über gemeinsame Planung in allen Bereichen der Spitalversorgung. Thurgau.

[40] Gilardi F., Füglister K. (2008). Empirical Modeling of policy diffusion in Federal States: the dyadic approach. Swiss Political Sci Review.

[41] Shepherd M.E. (2025). The politics of rural hospital closures. Polit Behav.

[42] Holmes G.M., Slifkin R.T., Randolph R.K., Poley S. (2006). The effect of rural hospital closures on community economic health. Health Serv Res.

[43] Faessler L., Lalive R., Efferson C. (2024). How culture shapes choices related to fertility and mortality: causal evidence at the Swiss language border. Evolut Hum Sci.

[44] Observatory European, on Health Systems and Policies, Giulio de Belvis A, Meregaglia M, Morsella A, Adduci A, Perilli A (2022).

[45] https://www.bundesgesundheitsministerium.de/themen/krankenhaus/krankenhausreform/faq-krankenhausreform.html.

[46] (2025). Bundesministerium für Gesundheit. KHAG.

