# Supplementary material for: What drives regional variation in hospital reform implementation? Insights from more than a decade of Swiss hospital planning
Source: Health Policy Open. 2026 Oct 14;11:100190. doi: 10.1016/j.hpopen.2026.100190 (PMC13694885; doi:10.1016/j.hpopen.2026.100190)

Supplementary material 1: Overview of different service-specific requirements from Zurich's 2025 definition [1] **(an English translation)**

For hospital planning, cantons have general and service-specific requirements. These are publicly available on the canton's website. Below, as an example, are some service-specific requirements translated from ZH's 2025 hospital planning list [1].

Level of availability of medical specialists

Zurich outlines four levels of availability, ranging from reachability within one hour (Level 1) to on-site presence within 15 minutes for obstetrics (Level 4).

Table 1: Definition of levels of availability of medical specialists according to Zurich HCP documents.

ICU Levels	Description
Level 1	The medical specialist must be reachable within 1 hour, or the patient must be transferred within 1 hour.
Level 2	The medical specialist must be available at all times. A diagnostic or therapeutic intervention must be possible within 1 hour; exceptionally, this may be ensured by other means.
Level 3	The medical specialist must be available at all times. A diagnostic or therapeutic intervention must be possible within 30 minutes.
Level 4 (Obstetrics)	The medical specialist must be on-site within 15 minutes of childbirth.

Emergency care

The Zurich model distinguishes four levels of emergency care, ranging from basic daytime availability of internal medicine and surgical staff (Level 1) to 24/7 on-site presence of obstetric specialists within 10 minutes (Level 4).

Table 2: Definition of levels of emergency care requirements according to Zurich HCP documents.

Emergency level	Description of level
1	Monday–Friday, 8 am to 5 pm: Physicians with certification in General Internal Medicine and Surgery must be available for the emergency department (multi-functional hospital staff deployment).<br>Monday–Friday, 5 pm to 8 am, and 24/7 on weekends and public holidays: Residents in General Internal Medicine and Surgery must be available for the emergency department. If medically necessary, a board-certified physician in General Internal Medicine and a board-certified physician in Anaesthesiology or Surgery must be available on-site within 15 minutes.
2	Monday–Friday, 8 am to 5 pm: Physicians with board certification in General Internal Medicine and Surgery must be available as the first priority for the emergency department. They are not permitted to be concurrently assigned to operating

	theatres.<br>Monday–Friday, 5 pm to 8 am, and 24/7 on weekends and public holidays: Same requirements as for Level 1.
3	Monday–Friday, 8 am to 11 pm: Same requirements as Level 2.<br>Monday–Friday, 11 pm to 8 am, and 24/7 on weekends and public holidays: Residents in General Internal Medicine and Surgery must be available as first priority. If medically necessary, they must be at the emergency department within 5 minutes. One of these physicians must be a resident in the second half of their specialist training.<br><br>Additionally, at least one physician with board certification in Surgery must be available on-site within 15 minutes (they may also be involved in surgery only for emergency cases), and a physician with board certification in General Internal Medicine must be available on-site within 30 minutes.<br>If medically required, a board-certified physician in Anaesthesiology (in-house) or Intensive Care Medicine must be available. For paediatric hospitals, a board-certified paediatrician must be available on-site from Monday to Sunday, 11 pm to 8 am, within 45 minutes.
4 (Obstetrics)	Monday–Sunday, 24 hours/day: A physician with board certification in Gynaecology and Obstetrics must be available on-site for the delivery unit within 10 minutes.<br>The emergency delivery case must be managed within 15 minutes; this is defined as the time between the decision for Caesarean section and the birth.<br>If medically necessary, a board-certified anaesthesiologist (in-house) or a midwife (on-site) must be present.

Intensive Care Unit (ICU) (Zurich's 2025 definition)

The Zurich model defines three levels of ICU, from observation units (Level 1) to certified training institutions (Level 3), based on criteria set by the Swiss Society of Intensive Care Medicine (SGI) [1].

Table 3: Definition of intensive care unit (ICU) levels according to the Zurich HCP documents.

ICU Levels	Description
1 - Intermediate Care Unit	Further requirements can be found in the annex "Additional Service-Specific Requirements" of the hospital list.
Level 2 – ICU certified by the SGI	The certification guidelines of intensive care units as defined by the Swiss Society of Intensive Care Medicine (SGI), including the quality criteria in Annex I, must be fulfilled.
Level 3 – ICU certified as SGI Training Centre	The guidelines for recognition as a training centre by the SGI, including the quality criteria in Annex I, must be met.<br>Additionally, the following FMH (Swiss Medical Association) criteria for a Category A training centre must be fulfilled:<br>At least 3,000 patient care days per year and at least 24,000 ventilation hours according to DRG per year.

Sources: 1. Gesundheitsdirektion Kanton Zürich. Zürcher Spitalplanung 2023. 2022.

Supplementary Material 2: Sources for cantonal HP

Table 4. Sources of current hospital lists and SPLG requirements.

Canton	Hospital List	Requirements of SPLGs
**AG**	https://www.ag.ch/media/kanton-aargau/dgs/dokumente/gesundheit/gesundheitsversorgung/spitaeler-kliniken/241218-spitalliste-2025-akutsomatik-rrb-nr-2024-001610.pdf	https://www.ag.ch/media/kanton-aargau/dgs/dokumente/gesundheit/gesundheitsversorgung/spitaeler-kliniken/d-anhang-5-zur-rrb-nr-2024-001212-2.pdf
**AI**	https://www.ai.ch/themen/gesundheit-alter-und-soziales/gesundheitsversorgung/spitallisten-und-spitalfinanzierung/akutsomatik/dokumente-spitalplanung-akutsomatik-araisg-per-1-januar-2025/spitalliste-akutsomatik-araisg-2024-2-per-januar-2025.pdf/download	https://www.ai.ch/themen/gesundheit-alter-und-soziales/gesundheitsversorgung/spitallisten-und-spitalfinanzierung/akutsomatik/dokumente-spitalplanung-akutsomatik-araisg-per-1-januar-2025/leistungsspezifische-anforderungen-an-die-listenspitaeler-araisg-2024.pdf/download
**AR**	https://ar.ch/fileadmin/user_upload/Departement_Gesundheit_Soziales/Amt_fuer_Gesundheit/03_Spitalversorgung/01_Spitalplanung/Spitalliste/Spitalliste_Akutsomatik_ARAISG_2024.2.pdf	https://ar.ch/fileadmin/user_upload/Departement_Gesundheit_Soziales/Amt_fuer_Gesundheit/03_Spitalversorgung/01_Spitalplanung/Spitalliste/Spitalliste_Akutsomatik_ARAISG_2024.2.pdf
**BL**	https://bl-api.webcloud7.ch/politik-und-behorden/direktionen/volkswirtschafts-und-gesundheitsdirektion/amt-fur-gesundheit/spitaeler-und-therapieeinrichtungen/spitallisten/dateien-spitallisten/spitalliste-akutsomatik.pdf	Not publicly available. BL uses ZH2023 (Information obtained from the cantonal representative via email).
**BS**	https://media.bs.ch/original_file/42bc8aaef34e2d5ff5282b4cee18b84f35781781/spitalliste-akutsomatik-25-01.pdf	Not publicly available. BS uses ZH2023 (Information obtained from the cantonal representative via email).
**BE**	https://www.gsi.be.ch/content/dam/gsi/dokumente-bilder/de/themen/gesundheit/gesundheitsversorger/spitaeler-psychiatrie-rehabilitation/spitalliste/sl-akutsomatik-2019-laufende-aenderungen-01-07-2025.pdf	https://www.gsi.be.ch/content/dam/gsi/dokumente-bilder/de/themen/gesundheit/gesundheitsversorger/spitaeler-psychiatrie-rehabilitation/spitalliste/splg-systematik-akutsomatik-be-version-2025.pdf
**FR**	https://bdlf.fr.ch/frontend/annex_document_dictionaries/5302	Uses ZH2025 for new planning cycle.
**GE**	https://www.ge.ch/document/27217/telechargergenf	Not publicly available. GE uses ZH2025 (Information obtained from the cantonal representative via email).
**GL**	Glarner_Spitalliste_2012_Akutsomatik_V2015.2.pdf	https://www.gl.ch/public/upload/assets/4413/Anhang_Spitalliste_-_Leistungsspezifische_Anforderungen.pdf?fp=1

**GR**	https://www.gr.ch/DE/institutionen/verwaltung/djsg/ga/InstitutionenGesundeitswesens/Spitaeler/Dok%20Spitler/Spitalliste_Akutsomatik.pdf	Not publicly available. GR uses ZH2014 (Information obtained from the cantonal representative via email).
**JU**	https://www.jura.ch/Htdocs/Files/v/c680f757270a90ffdb65bbd8c8236a81823a183880bd7286a0795e103b3a0066.pdf/Liste-hospitaliere-2023.pdf?download=1	Not publicly available. JU uses ZH2025 (Information obtained from the cantonal representative via email).
**LU**	https://gesundheit.lu.ch/-/media/Gesundheit/Dokumente/Gesundheitsversorgung/Spitalliste/Spitalliste_vom_22032016_mit_nderungen_23012024_inklSt.pdf?rev=0954de434e304a59b76e4034e82a3519	Not publicly available. LU uses ZH2019 (Information obtained from the cantonal representative via email).
**NE**	https://www.ne.ch/autorites/DFS/SCSP/Documents/ListeHospitaliereNE_2016-22_01092023.pdf	https://www.ne.ch/autorites/DFS/SCSP/hopitaux-institutions/Documents/PlanifHospitalRapportCondCadres.pdf
**NW**	https://www.nw.ch/_docn/418336/Spitalliste_2017-12.pdf	Not existent.
**OW**	https://gdb.ow.ch/app/de/texts_of_law/830.511/versions/1428	Not existent.
**SG**	https://www.sg.ch/gesundheit-soziales/gesundheit/gesundheitsversorgung--spitaeler/spitalplanung-spitalliste/_jcr_content/Par/sgch_accordion_list/AccordionListPar/sgch_accordion_458606438/AccordionPar/sgch_downloadlist/DownloadListPar/sgch_download.ocFile/Spitalliste_Akutsomatik_SG_2024_I_Nachtrag.xlsx	https://www.sg.ch/gesundheit-soziales/gesundheit/gesundheitsversorgung--spitaeler/spitalplanung-spitalliste/_jcr_content/Par/sgch_accordion_list/AccordionListPar/sgch_accordion_458606438/AccordionPar/sgch_downloadlist/DownloadListPar/sgch_download_871643544.ocFile/SPLG_2025.ARAISG01.xlsx
**SH**	https://sh.ch/CMS/get/file/b6df40ca-fe4d-4dd0-abbf-7357926ac331	Not publicly available. SH uses ZH2025 (Information obtained from the cantonal representative via email).
**SO**	https://so.ch/fileadmin/internet/ddi/ddi-gesa/PDF/Behandlung_und_Pflege/Spitalliste_Akut_2024_07_01.pdf	Not publicly available. SO uses ZH2023 (Information obtained from the cantonal representative via email).
**SZ**	https://www.sz.ch/public/upload/assets/67681/Schwyzer_Spitalliste_2025_Akutsomatik_Stand_1-Juli-2025.pdf?fp=8	Not publicly available. SZ uses ZH2025 (Information obtained from the cantonal representative via email).
**TG**	https://gesundheit.tg.ch/public/upload/assets/138352/Thurgauer_Spitalliste_Akutsomatik_Version_2023.1.pdf?fp=2	Not publicly available. TG uses ZH2023 (Information obtained from the cantonal representative via email).
**TI**	https://www4.ti.ch/fileadmin/DSS/DSP/AGS/Elenco_ospedaliero_stato_al_22.12.2021.pdf	Not publicly available. TI uses ZH2012 (Information obtained from the cantonal representative via email).
**UR**	https://rechtsbuch.ur.ch/lexoverview-home/lex-20_3235?effective-from=20230101	Not existent.

Supplementary material 3: Consolidated Criteria for Reporting Qualitative Research (COREQ)

No. Item	Guide questions/description	Reported on Page #
**Domain 1: Research team and reflexivity**		
Personal Characteristics		
1. Interviewer/facilitator	Which author/s conducted the interview or focus group?	Page 9
2. Credentials	What were the researcher's credentials? E.g. PhD, MD	See blow the description of the research team & reflexivity
3. Occupation	What was their occupation at the time of the study?	See blow the description of the research team & reflexivity
4. Gender	Was the researcher male or female?	See blow the description of the research team & reflexivity
5. Experience and training	What experience or training did the researcher have?	See blow the description of the research team & reflexivity
Relationship with participants		
6. Relationship established	Was a relationship established prior to study commencement?	See blow the description of the research team & reflexivity
7. Participant knowledge of the interviewer	What did the participants know about the researcher? e.g. personal goals, reasons for doing the research	See blow the description of the research team & reflexivity
8. Interviewer characteristics	What characteristics were reported about the interviewer/facilitator? e.g. Bias, assumptions, reasons and interests in the research topic	See blow the description of the research team & reflexivity
**Domain 2: study design**		
Theoretical framework		
9. Methodological orientation and Theory	What methodological orientation was stated to underpin the study? e.g. grounded theory, discourse analysis, ethnography, phenomenology, content analysis	Page 10
Participant selection		
10. Sampling	How were participants selected? e.g. purposive, convenience, consecutive, snowball	Page 9-10

11. Method of approach	How were participants approached? e.g. face-to-face, telephone, mail, email	Page 9-10
12. Sample size	How many participants were in the study?	Page 9-10
13. Non-participation	How many people refused to participate or dropped out? Reasons?	Page 9-10
Setting		
14. Setting of data collection	Where was the data collected? e.g. home, clinic, workplace	Page 9-10<br>.
15. Presence of non-participants	Was anyone else present besides the participants and researchers?	See blow the description of the research team & reflexivity
16. Description of sample	What are the important characteristics of the sample? e.g. demographic data, date	Page 6, supplementary material 4
Data collection		
17. Interview guide	Were questions, prompts, guides provided by the authors? Was it pilot tested?	supplementary material 5 and 6
18. Repeat interviews	Were repeat interviews carried out? If yes, how many?	No repeat interviews were carried out
19. Audio/visual recording	Did the research use audio or visual recording to collect the data?	Page 9
20. Field notes	Were field notes made during and/or after the interview or focus group?	See blow the description of the research team & reflexivity
21. Duration	What was the duration of the interviews or focus group?	Page 9
22. Data saturation	Was data saturation discussed?	The 25 interviews comprised two analytically distinct groups. The first group consisted of five expert interviews with national-level stakeholders, conducted to get non-publicly available information on the reform's design and intended implementation, and to triangulate findings from the cantonal document analysis. For this group, data saturation was not the primary criterion, as the interviews targeted a defined set of actors with specific institutional knowledge.

23. Transcripts returned	Were transcripts returned to participants for comment and/or correction?	Page 9
**Domain 3: analysis and findings**		
Data analysis		
24. Number of data coders	How many data coders coded the data?	Page 10
25. Description of the coding tree	Did authors provide a description of the coding tree?	supplementary material 7
26. Derivation of themes	Were themes identified in advance or derived from the data?	Page 9
27. Software	What software, if applicable, was used to manage the data?	ATLAS.ti (version 25.0.1)
28. Participant checking	Did participants provide feedback on the findings?	Page 9
Reporting		
29. Quotations presented	Were participant quotations presented to illustrate the themes/findings? Was each quotation identified? e.g. participant number	Page 11-27
30. Data and findings consistent	Was there consistency between the data presented and the findings?	Page 11-27
31. Clarity of major themes	Were major themes clearly presented in the findings?	Page 11-27
32. Clarity of minor themes	Is there a description of diverse cases or discussion of minor themes?	Page 11-27

Research team & reflexivity

CW (female), a second-year PhD student, CL (female), a first-year PhD student, and KL (female), a master’s student, conducted all interviews with cantonal representatives and experts. For the French-speaking cantons, a trained second-year PhD student (male) who is not a co-author assisted CW or CS in conducting the French interviews. DB (female), a second-year PhD student, supported the development of the interview guide and contributed to the interpretation of findings. DE and JV (both male), postdoctoral researchers and project leads, as well as AG (male), a full professor, contributed to the study design, the validation of the interview guide and interpretation of findings.

All researchers had at least basic training in qualitative methods, with CW having completed formal coursework and leading the qualitative coding process. CW and CL coded all interviews independently using a combination of deductive and inductive approaches.

No prior relationships existed between researchers and participants. Before each interview, participants received the interview guide and a confidentiality agreement. Prior to the interviews, participants were informed of the voluntary nature of their involvement and their right to withdraw at any time without consequence. At the beginning of each interview, interviewers introduced themselves, explained the study aims, and outlined their roles. General academic background was shared for transparency, while personal motivations were not disclosed.

Supplementary material 4: Overview of interviews

Description of experts

Table 5 provides an overview of the five expert interviews conducted, including brief descriptions of the experts, who remain anonymous.

Table 5. Overview of experts and their designation in the text

Designation	Role; Organisational context; HP-relevance	Sector	Years of experience in HP	Gender
Expert 1	Consultant; specialized in hospital planning; directly involved in the development of the SPLG methodology	Private	>15 years	Male
Expert 2	Former head of a cantonal health department; overseeing HP in a major canton during the KVG reform.	Public	>20 years	Male
Expert 3	Consultant specialised in healthcare strategy; advised multiple cantons on HP	Private	>10 years	Male
Expert 4	Representative of a national association representing private hospitals; represents private hospital interests in HP policy discussions at the national level	Private (association)	>10 years	Male
Expert 5	Representative for the intercantonal coordination body	Public (intercantonal)	>10 years	Female

Description of cantonal representatives

A total of 20 cantonal representatives participated in semi-structured interviews. To protect participant anonymity, which was considered essential for enabling open and candid responses on politically sensitive topics such as minimum volume requirements, individual participants are not identified by name, canton, or specific job title.

Participants represented cantonal health authorities across all major linguistic regions of Switzerland, including German-speaking (n=14), French-speaking (n=5), and Italian-speaking (n=1) cantons. Cantons of varying size, urbanisation, and hospital landscape were represented, reflecting the structural diversity of the Swiss cantonal system.

In terms of professional role, participants held senior positions within their respective cantonal health departments, including heads of health offices and senior officers responsible for hospital planning. Years of experience in their role ranged from 6 months to over 20 years. In terms of gender distribution, 5 females and 15 males participated in the interviews.

Supplementary material 5: Interview Guide Experts

While the preparation and introduction to the interview remained the same as for the cantonal representatives, the questions were slightly adjusted to achieve a more national perspective.

a) General Introduction

1. Can you tell me something about hospital capacity planning in Switzerland in general?
2. What do you think are the biggest challenges of hospital capacity planning in Switzerland?
3. How is the Swiss population changing demographically, and what impact does this development have on future hospital planning?

b) Comparison with the Zurich Model

1. Do you think other cantons have adopted the Zurich model as is, or were there delays or resistance? If so, where and to what extent?
2. Are there significant differences between the so-called Zurich model of SPLGs and the models in other cantons (especially German-speaking, French-speaking, and Italian-speaking)? If so, what are they?
3. Do you think the current reform is sustainable for the future? How do you generally assess the future of the healthcare landscape, focusing on national, cantonal, or regional-cooperative planning?
4. What is your opinion on intercantonal hospital capacity planning? Are there any existing or planned projects? If so, in which cantons?

c) Evaluation

1. Has the change in hospital capacity planning had an impact on:
   a) The economic efficiency of hospitals?
   b) The quality of hospitals?
   c) The consolidation of hospitals?

d) Future Perspectives

1. What adjustments would you recommend improving the current model of hospital capacity planning?

Supplementary material 6: Interview Guide Cantons

The document analysis and research questions provided the basis for developing the interview guides. The guides were created in German and subsequently translated into French by a bilingual native speaker. The following interview guide was shared beforehand with all participants in German or French, depending on the region. It has been translated to English for this manuscript.

a) Preparation and introduction

- Welcome: Express gratitude for participation.
- Explanation of context: SNF project The Swiss hospital capacity planning: An empirical evaluation of its impact on hospital service delivery, quality and costs.
- Objective: Discussions with the health departments of various cantons about their perceptions and implementation.
- Duration: 35-45 minutes.
- Emphasise voluntary participation: You are not obliged to answer a question if you do not want to or cannot.
- Confidentiality: The interview will be recorded for transcription and analysis. Your information will be kept confidential. Your answers will be anonymised and will not be published under your name. No one except you and us will know what was said during the interview.
- Declaration of consent: Verbal declaration and presentation of a written document for signature (sent in advance).
- Questions: Opportunity to ask questions.

b) Interview questions - Start

1. Can you tell me about hospital planning in Canton X?
2. How did you perceive the implementation of the reform (KVG revision, which came into force in 2009) of hospital capacity planning in Switzerland?
3. In your opinion, what are the biggest challenges facing hospital capacity planning in Switzerland?

4. Hospital landscape in Canton X: How many public and private hospitals are there in Canton X? How many hospitals are on the list, and how many are contractually bound? What distinguishes the hospital landscape in X from other cantons?

c) Timetable for implementing the reform

1. When was the reform officially introduced in Canton X (year/month)?

2. What did hospital planning look like before the reform was introduced?

3. Have there been any adjustments since the reform?

4. What factors influenced the speed of reform implementation in Canton X?

d) Hospital capacity planning in Canton X

1. How did Canton X implement the revision of the KVG and the reform in its hospital planning?

2. What is the legal basis for hospital capacity planning in Canton X?

3. Did Canton X base its implementation on the 'Zurich model' and structure its hospital capacity planning in a similar way?

4. Are there any differences between the Zurich model and the version implemented in Canton X? If so, what are they?

- Financing
- SPLG, requirements
- Quality

5. How regulated is Canton X compared to other cantons in terms of hospital capacity planning on a scale of 1 to 5 (little to highly regulated)?

6. How are need-for-care analyses calculated and carried out? Have local conditions (e.g. demographic factors such as population size and age or topographical criteria such as rural vs. urban areas) influenced the implementation of the reform?

7. How do patient flows work in Canton X?

8. How does Canton X position itself in relation to intercantonal hospital capacity planning?

e) Assessment

1. Has the revision of the KVG (hospital capacity planning) had an impact on the following aspects:

- the profitability of hospitals?

- the quality of hospitals?
- the consolidation of hospitals?

f) centralisation of service mandates

1. Do you think that the reform of hospital capacity planning has led to a centralisation of service mandates in Canton X? If so, in what way?

2. Are there any regions, hospitals or service groups that immediately come to mind when you think of centralisation?

g) Future prospects

1. What adjustments would you recommend improving the current hospital planning model in Canton X / Switzerland?

Supplementary material 7: Coding framework

Figure 1: Qualitative content analysis using the framework approach

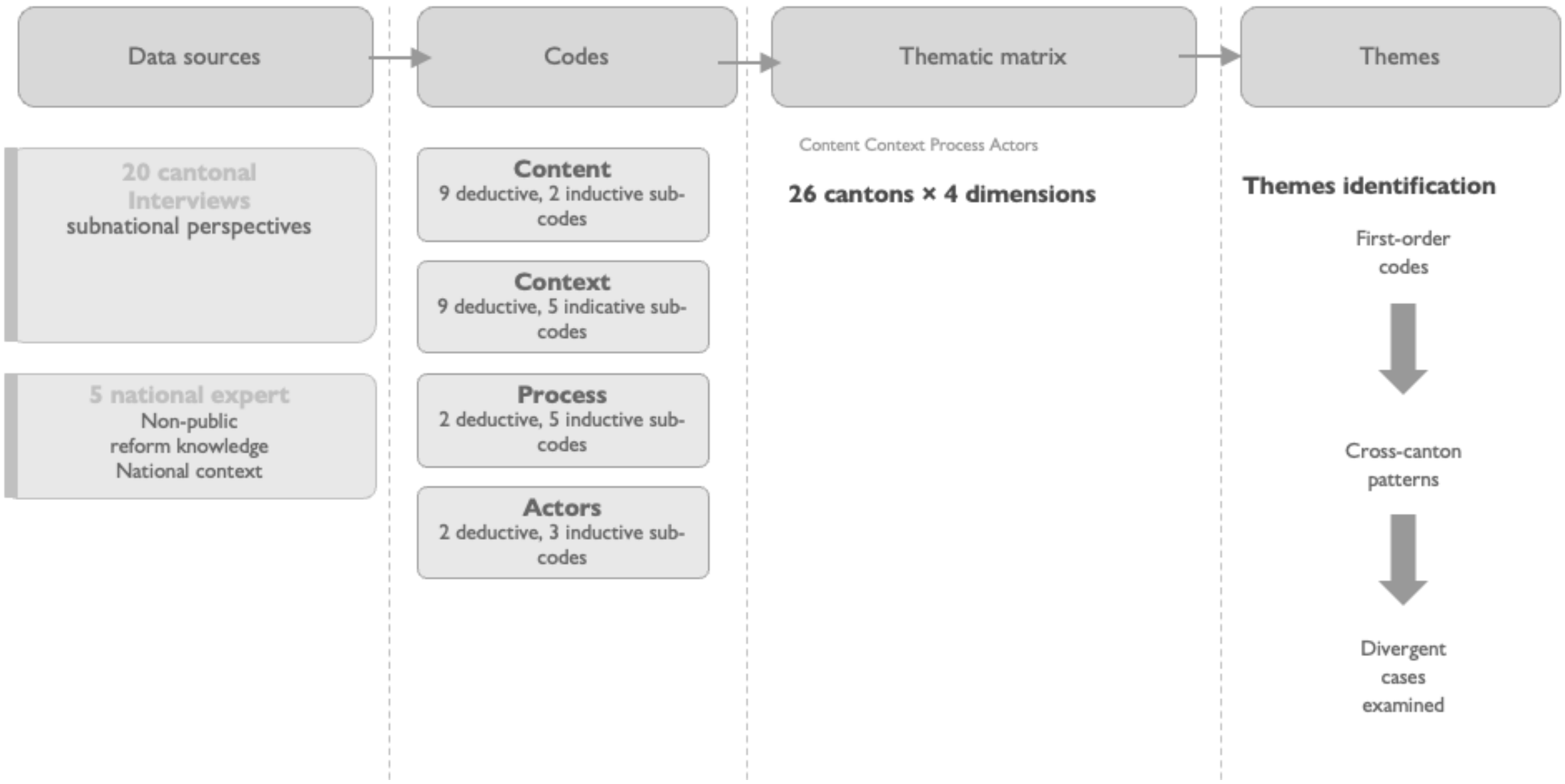

Supplement: Supplementary file 1 [file mmc1.pdf]
